# Exercise‐induced eosinophil responses: Normal cell counts with a marked decrease in responsiveness

**DOI:** 10.1002/clt2.12314

**Published:** 2023-11-16

**Authors:** Bernard N. Jukema, Thomas C. Pelgrim, Sylvan L. J. E. Janssen, Thijs M. H. Eijsvogels, Alma Mingels, Wim Vroemen, Nienke Vrisekoop, Leo Koenderman

**Affiliations:** ^1^ Department of Respiratory Medicine University Medical Center Utrecht Utrecht University Utrecht The Netherlands; ^2^ Center for Translational Immunology University Medical Center Utrecht Utrecht University Utrecht The Netherlands; ^3^ Department of Medical BioSciences Exercise Physiology Research Group Radboud University Medical Center Nijmegen The Netherlands; ^4^ Central Diagnostic Laboratory Maastricht University Medical Center Maastricht The Netherlands

To the Editor,

Type II inflammation is characterized by elevated blood eosinophils which makes these cells an important diagnostic and treatment target in, for instance, severe asthma. Therefore, blood eosinophil numbers are a main inclusion criterion for many clinical studies that have investigated the treatment of eosinophilic asthma with, for example, anti‐IL5(Rα).^1^ However, there is no consensus on cut‐off values for blood eosinophils at inclusion as evidenced by a high variability between studies, ranging from 150 to 400 cells/μL. Moreover, the range of blood eosinophils in a healthy population, without confounding factors for increased blood eosinophils, is 30–330 cells/μL in males and 30–310 cells/μL in females.^2^ This implies that the cut‐off values used for clinical studies greatly overlap with blood eosinophil counts that are found in the healthy population. This inherently poses a problem as eosinophil blood counts seem to be inadequate to use for diagnosing eosinophilic diseases other than hypereosinophilia (>1500 cells/μL).

This overlap in eosinophil counts between patients and the healthy population limits the application of eosinophil numbers for discriminating between health and several inflammatory diseases. Eosinophil activation status ex vivo has already been investigated primarily with regard to asthma phenotypes,^3^ but this study did not account for the effects of ex vivo activation caused by the manipulation of cells during sample work‐up procedures. So, at least part of the activation phenotype might have been caused by enhanced sensitivity for ex vivo activation under these inflammatory conditions. Thus, a more promising approach in diagnosing eosinophilic disease would be to combine eosinophil numbers with their activation status under controlled conditions with minimal ex vivo manipulation.^4^ This could improve eosinophil diagnostics, particularly in situations with (relative) eosinopenia. Unfortunately, there is surprisingly little evidence that blood eosinophil *counts* correlate with their activation status and/or responsiveness in vivo. Apart from activation ex vivo,^5^ this lack of correlation can also be caused by homing of activated cells to the lung leaving behind non‐activated cells in the blood.^4^, ^6^

Most studies on eosinophil activation in vivo have been performed in the context of T‐2 diseases. These studies imply that eosinophil activation in vivo is mainly driven by T‐2 cytokines such as IL‐5. Surprisingly little is known about eosinophil activation in vivo in donors without inflammatory diseases. Therefore, we designed this study on eosinophil activation in healthy individuals. Minimal ex vivo activation was achieved by analyzing blood eosinophils directly after venipuncture with a fast, automated, point‐of‐care, mobile flow cytometer (AQUIOS CL, Beckman Coulter). For the study of eosinophil activation in vivo, we applied exercise as a model to modulate eosinophil numbers in a healthy setting.^7^ We tested the hypothesis that eosinophil blood counts correlate with their activation status and their responsiveness to formyl peptides in a cohort of 35 long‐distance runners participating in a mass‐participation trail run (22, 29 or 43 km). Venous blood samples were collected from the athletes before, directly after and 24 h after exercise. The flow cytometer performed two parallel analyses of each sample: in the absence and presence of the formyl‐peptide N‐formyl‐norleucyl‐leucyl‐phenylalanine (fNLF; 10 μM). This approach discriminated in vivo activation (increased expression of activation markers in the absence of a stimulus) from pre‐activation or priming (increased susceptibility for activation with fNLF visualized by expression of the same markers). This point‐of‐care approach allowed us to perform flow cytometry analyses of the blood samples of the 35 athletes on the three different time points with a median time of 30 min between venipuncture and completion of the analysis. This is markedly faster than traditional flow cytometry work‐up and analysis procedures. The eosinophil activation status was assessed by combining automated flow cytometry with a five‐dimensional algorithm (FlowSOM)‐based gating as was previously described by Jukema et al.^8^

An acute leukocytosis with eosinopenia was present directly post‐exercise, which is in agreement with previous research.^7^ These numbers normalized 24 h after exercise (Figure 1A,C). Compared to before exercise, eosinophils showed a more activated phenotype (increased CD11b and decreased CD62L expression) directly after exercise, which normalized within 24 h. In marked contrast to acute inflammation, such as caused by severe acute respiratory syndrome coronavirus 2 infection,^9^ this eosinopenia directly after exercise did not lead to refractoriness to fNLF‐stimulation. However, after the normalization of eosinophil counts 24 h after exercise, the cells did become refractory for activation by fNLF (Figure 1B,C). This clearly showed a complete dissociation between blood eosinophil numbers and their pre‐activation status.

**FIGURE 1:**
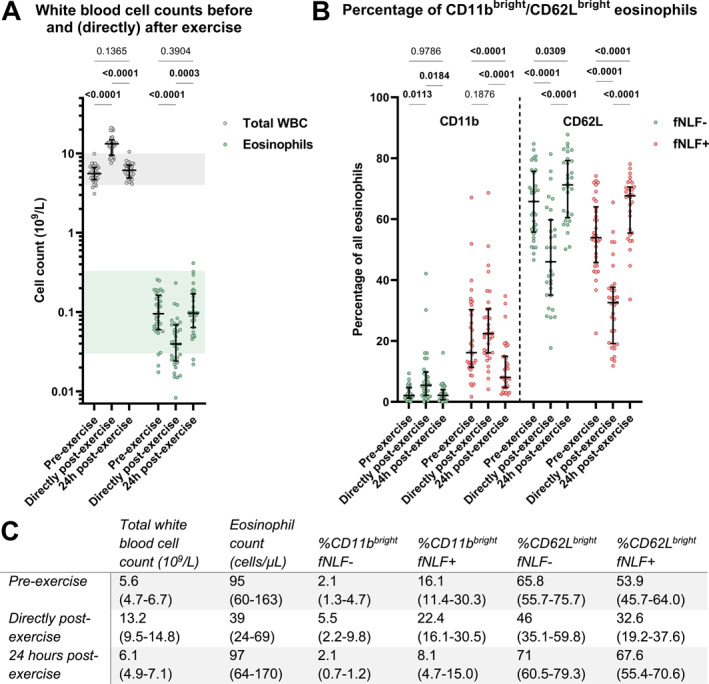
(A) Total white blood cell and blood eosinophil counts in trail run participants at three different time points around a trail run. The shaded areas indicate general reference values for total white blood cell (WBC; 4–10 × 10^9^/L) and eosinophil counts (0.03–0.33 × 10^9^/L). (B) The percentage of CD11b^bright^ and CD62L^bright^ blood eosinophils at three different time points around the trail run. fNLF−/fNLF+ samples were measured in the absence/presence of the formylpeptide (10 μM). Statistical significance was determined with a mixed‐effects model with Geisser‐Greenhouse correction and Tukey's multiple comparisons test. (C) Median (interquartile range) values of the data in panels (A and B).

Our results illustrate that the eosinophil blood compartment is not adequately characterized by solely counting cell numbers (‘quantity’) as normalized numbers do not necessarily reflect normalization of their (pre‐)activation status (‘quality’). This finding is not limited to measuring the state of type II immunity in eosinophilic disease, but also applies to other infectious/inflammatory conditions and non‐pathological settings such as exercise. Our data call for a re‐evaluation of using blood eosinophil counts as a sufficient representation of the eosinophil compartment's state. Until recently, determining the activation status of the eosinophil compartment was complicated by ex vivo artifacts already starting at the moment of venipuncture. Now with the availability of fast, automated, point‐of‐care flow cytometry, it is feasible to measure both the *quantity* and *quality* of eosinophils in a wide scope of health and disease settings.

## AUTHOR CONTRIBUTIONS

*Conceptualization*: Bernard N. Jukema, Sylvan L. J. E. Janssen, Alma Mingels, Wim Vroemen, Thijs M. H. Eijsvogels, Leo Koenderman. *Methodology*: Bernard N. Jukema, Sylvan L. J. E. Janssen, Thijs M. H. Eijsvogels, Leo Koenderman. *Data analysis*: Bernard N. Jukema. *Visualization*: Bernard N. Jukema. *First draft writing*: Bernard N. Jukema, Leo Koenderman. *Revision and approval of final paper*: all authors.

## CONFLICT OF INTEREST STATEMENT

The AQUIOS CL® ‘Load & Go’ flow cytometer is provided by the company Beckman Coulter Life Sciences, Miami, FL, USA. This company had no role in the study's design, the data analysis, the article's preparation, or the decision to submit the article for publication. The authors declare that the research was conducted in the absence of any commercial or financial relationships that could be construed as a potential conflict of interest.

## FUNDING INFORMATION

Radboud University Medical Center; Academic Alliance Fund.

## Data Availability

The data that support the findings of this study are available from the corresponding author upon reasonable request.
